# FOOTWEAR PURCHASING PATTERN DURING THE COVID-19 PANDEMIC: A CROSS-SECTIONAL STUDY

**DOI:** 10.1590/1413-785220253302e285417

**Published:** 2025-06-02

**Authors:** Fernando Gonzalez Correa, Lucas Plens de Britto Costa, Lucas Furtado da Fonseca, Leonardo Fernandez Maringolo, Caio Augusto Souza Nery, Tania Szejnfeld Mann

**Affiliations:** 1 Universidade Federal de São Paulo, Departamento de Ortopedia e Traumatologia, Centro de Traumatologia Esportiva, São Paulo, SP, Brazil.

**Keywords:** Habits, Shoes, e-Commerce, COVID-19, Hábitos, Sapatos, Comércio Eletrônico, COVID-19

## Abstract

**Introduction:**

The COVID-19 pandemic boosted the increase in online sales, however there is a lack of research on the shoe purchasing pattern among the Brazilian population.

**Objective:**

To investigate the shoe purchasing pattern.

**Method:**

This cross-sectional study comprises individuals from a Foot and Ankle Outpatient Clinic. A total of 500 individuals were invited to respond to a digital form using the Google Forms tool. Stata® software version 13.0 was used for statistical analyses.

**Results:**

433 individuals were included, 86.37% of whom were women. Purchasing shoes online was reported by 44.34% (n=192/433) of individuals, and 13.39% (n=58/433) said that the choice of digital platforms was due to the practicality of purchasing online. There were differences in the shoe purchasing pattern according to female and male gender (p<0.001).

**Conclusion:**

Although most of the individuals surveyed are not used to buying shoes online, most purchases were made by women, mainly aiming for comfort and beauty. This study highlights the need to develop tools that help people purchase shoes online. ***Level of Evidence I; High-quality prospective study.***

## RESUMO

**Introdução:**

A pandemia de COVID-19 impulsionou o aumento das vendas on-line, contudo há uma carência de pesquisas sobre o padrão de compra de calçados na população brasileira.

**Objetivo:**

Investigar o padrão de compra de calçados.

**Método:**

Trata-se de um estudo transversal, composto por indivíduos de um Ambulatório de Pé e Tornozelo. Um total de 500 indivíduos foram convidados a responder um formulário próprio e digital através da ferramenta Google Forms. O software Stata®, versão 13.0, foi utilizado para as análises estatísticas.

**Resultados:**

Foram incluídos 433 indivíduos, sendo 86,37% mulheres. A compra de calçados pela internet foi relatada por 44,34% (n=192/433) dos indivíduos e 13,39% (n=58/433) disseram que a escolha de plataformas digitais deve-se a praticidade ao comprar on-line. Houve diferenças no padrão de compra de calçados conforme o gênero feminino e masculino (p<0,001).

**Conclusão:**

Embora grande parte dos indivíduos pesquisados não estejam acostumados a comprar sapatos online, a maior parte das compras foram realizadas pelas mulheres, visando principalmente o conforto e a beleza. Este estudo destaca a necessidade de desenvolvimento ferramentas que auxiliem para compra on-line de calçados. ***Nível de Evidência I; Estudo prospectivo de alta qualidade.***

## INTRODUCTION

Footwear is the oldest fashion accessory and is considered a means of protecting the feet.^1–3^ The fit of footwear is essential to ensure comfort and prevent or treat orthopedic pathologies affecting the lower limbs and spine.^2,4–7^

Shopping in physical stores allows you to choose a better shoe fit and comfort. However, e-commerce, mainly driven by the COVID-19 pandemic,^8^ has made buying shoes online increasingly common. The increase in online research into the fit of footwear, especially for children, has been reported in the literature,^5,9^ but there is a lack of studies exploring the footwear purchasing patterns of adults in the Brazilian population. Therefore, this study aimed to investigate the footwear purchasing patterns of the Brazilian population.

## METHODS

This prospective cross-sectional study was carried out at the Orthopedics and Traumatology Department of the Universidade Federal de São Paulo – UNIFESP. It was approved by the UNIFESP Ethics Committee (CEP/UNIFESP/ n:0809P/2021) and complies with the Declaration of Helsinki.

A total of 500 individuals from the Orthopedics and Traumatology Outpatient Clinics at UNIFESP were invited to fill in a digital form using the Google Forms tool. The data was collected between January 2021 and January 2023.

Subjects of both sexes over 20 years of age were included. Individuals who refused to take part in the study, had pain, foot deformities, inflammatory diseases, comorbidities, or previous surgery were excluded.

Participants were asked for their age (20-40 years, 40-60 years and >60 years) and gender (female and male). We asked about the habit of shopping online (yes or no); the number of shoe purchases [once a week (50 shoes/year), once a month (12 shoes/year), 3 times a year (4 shoes/year), twice a year (2 shoes/year) and only on specific occasions] and what you consider when choosing shoes (beauty/design, comfort, recommendation from someone else (friend, salesperson) and price).

They were also asked about buying shoes online (yes or no); about the use of digital platforms to buy shoes (Don't trust that the shoes will fit; If they don't fit, it will be a pain to exchange them; Prefer the experience of going to the store to choose; Prefer the convenience of buying online) and the choice of brand/store [I always buy the same brand; I look for the shoe, regardless of the store; I search for models on the internet, then go and try them on in the store; I follow the recommendation of a doctor or a friend]. They were also asked if they used any tools, such as a virtual shoe fitting room, when buying shoes (yes or no).

Participants were also asked how often they bought shoes that hurt during use and had to stop wearing them and whether they used any tools, such as a virtual shoe fitting room, when buying shoes (yes or no). Would you like to use a tool to help you buy shoes that don't hurt (yes or no)? What would be the ideal way to receive advice on how to choose the best shoes (app, information leaflet, video lesson, or instruction manual)?

The last question was about how much you would be willing to pay for an app to take measurements and recommend suitable shoes (R$10/month; R$120 once; R$10/every time you wear it; or I don't want to pay anything, the shoe store has to offer it).

### Statistical analysis

Descriptive statistics were used to summarize the results. For descriptive analyses, we obtained absolute frequency (n) and percentage (%) values. Stata® software version 13.0 was used for the statistical analysis.

## RESULTS

**Figure 1:**
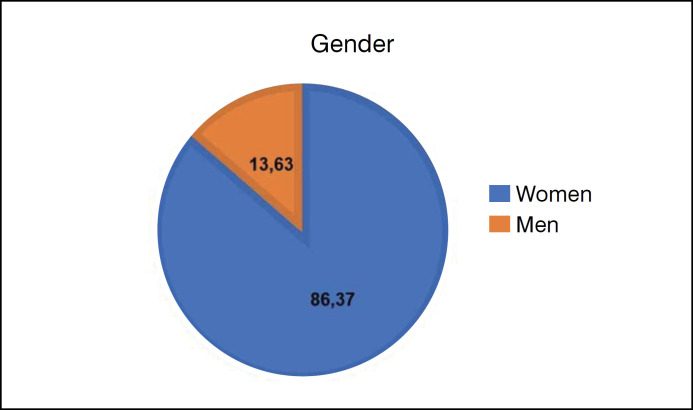
Gender graph of the survey participants: 86.37% women and 13.63% men.

**Figure 2:**
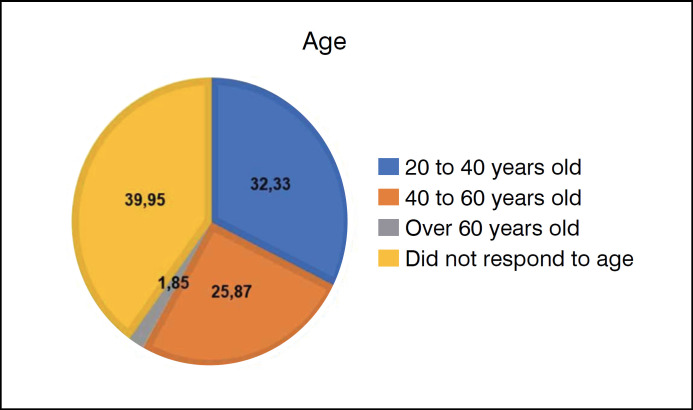
The age graph of the participants in the survey was 20-40 years, 32.33%; 40-60 years, 25.87%; over 60 years, 1.85%, and 39.95% did not choose to answer.

The sample consisted of 433 individuals (13.63% men and 86.37% women) (Figure 1). Of these, 32.33% (n=140) were aged between 20 and 40, 25.87% between 40 and 60, and 1.85% over 60. 39.95% of the participants chose not to answer their age (Figure 2).

**Figure 3:**
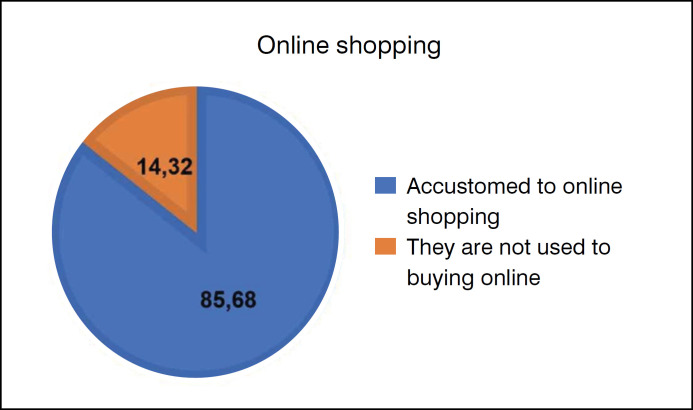
Online shopping chart: 85.68% of participants are used to shopping online, and 14.32% of participants are not used to shopping online.

More than half of the participants (n= 371/433 - 85.68%) reported that they are used to shopping online, and 44.34% (n=192/433) buy shoes through these channels (Figure 3).

Regarding the use of digital platforms to buy shoes, 16.16 % (n=70/433) don't trust that the shoes will fit; 34.64 % (n=150/433) think that if they don't fit, it will be a pain to exchange them; 35.79 % (n=155/433) prefer the experience of going to the store to choose the shoes; 13.39% (n=58/433) prefer the convenience of buying online. Most shoe purchases are made 3 times a year, 48.50% (n=210/433), and 2 times a year, 25.17% (n=109/433). 16.40% (n= 71/433) buy feathers on specific occasions.

**Figure 4:**
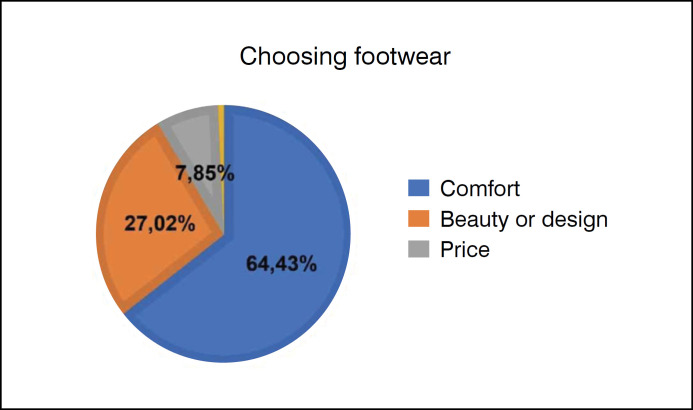
The age graph of the participants in the survey was 20-40 years, 32.33%; 40-60 years, 25.87%; over 60 years, 1.85%, and 39.95% did not choose to answer.

The choice of footwear is mainly based on comfort (64.43%, n=279/433), followed by the beauty or design of the shoes (27.02%, n=117/433) and price (7.85%, n=34/433) (Figure 4). Most of the participants reported that they rarely or never buy shoes that hurt their feet (48.04%, n=208/433, and 6.47%, n=28/433, respectively). When asked how they choose their shoe brand/store, 36.49% (n=158/433) said that they always buy shoes from the same brand, 9.93% (n=43/433) research the models on the internet and then try them on in the store and only 1.62% (n=7/433) follow the recommendation of a doctor or a friend. The rest, 51.96% (n=225/433), look for shoes on the Internet, regardless of the store. Most of the participants (93.76 % - 406/433) don't use digital fitting rooms or measurement tables to help them buy, although 88.68 % (n= 384/433) are interested in an online tool that could help them buy a shoe that doesn't hurt.

The preferred tool for guidance on how best to choose your shoes is apps on mobile devices (60.50 %, n= 262/433) and video lessons (16.62%, n=72/433). However, 50.35% (n=218/433) said they didn't want to pay for an app. Table 1 shows information on internet shopping patterns according to gender.

**Table 1:** Prevalence of footwear purchasing patterns according to gender (n = 433).

Variable	Gender		
	Female (n=374)	Male (n=59)	
	Prevalence n (%)		p
**Internet shopping habits**			**0.151**
Yes	324(93.27)	44(6.73)	
No	50(86.49)	15(13.51)	
**Buying shoes online**			**0.071**
Yes	171(95.65)	15(4.35)	
No	203(89.66)	44(10.34)	
**Use of a virtual shoe fitting room**			**1.000**
Yes	23(93.75)	2(6.25)	
No	351(92.21)	55(7.79)	

Fisher's exact test was used when the frequencies were less than five. p < 0.05 was considered statistically significant (bold).

## DISCUSSION

This study analyzed the footwear purchasing patterns of adults during the Covid-19 pandemic. As far as we know, this is the first study carried out with adults in São Paulo, Brazil. Among the contributions of this article, the following results stand out: there was a high prevalence of online shopping, and less than half of the participants reported shopping for shoes. The choice of digital platforms was mainly due to the convenience of buying online. In addition, most of the individuals in this survey buy shoes three times a year, and their choice is made with comfort in mind. The use of apps on mobile devices is the preferred tool to help guide people on how to choose their shoes better. Women make the majority of internet purchases and online shoe purchases.

In this study, we identified a high prevalence of internet shopping, mainly due to the convenience of buying online. The COVID-19 pandemic has resulted in a general increase in online shopping (e-shopping), especially for supermarket products. There has also been an increase in teleworking, teleconferencing, e-learning, and telehealth compared to the pre-COVID-19 period.^8,10^

Our findings indicate that there were few purchases of shoes online and that those who did buy them chose comfort in particular. We didn't find any studies that reported on adults buying shoes online. However, the literature indicated increased searches for online resources on the fit of children's footwear.^5^ The authors reported that choosing properly fitting footwear for children is challenging.^5^

The correct footwear fit is recognized as vital for comfort and mainly because using inappropriate or ill-fitting shoes is responsible for increased falls, ankle and foot injuries, and pain, especially in the elderly.^7^ Therefore, although online shopping has expanded to a variety of consumers, the choice and fit of footwear remains a challenge,^1^ especially when shopping online, and specific interventions for advice and education regarding footwear are needed.^6,7^

There were no differences in the pattern of footwear purchases according to gender. However, the literature indicates that men generally favor buying electronic products and women consume more clothing and accessories.^11^

The possible limitations of this study are the low number of men in our sample. Among the strengths of this research, we highlight the sample size, which is representative of the population. Thus, the findings of this study are essential to stimulate the development of online shopping tools to help fit shoes. The development of these tools could reduce dissatisfaction with purchases and provide greater comfort for buyers, and the increase in online sales will have a positive impact on our country's economy.

## CONCLUSION

Even with the great digitalization of shopping caused by the COVID-19 pandemic, many of the individuals surveyed are not used to buying shoes online. Even so, the majority of purchases are made by women, mainly for comfort and beauty. This study highlights the need to develop tools to help people buy shoes online.
